# Double Challenges for Scalp Health: A Review of the Pathogenesis and Treatment Strategies of Coexistence of Seborrheic Dermatitis and Androgenetic Alopecia

**DOI:** 10.1155/drp/8851971

**Published:** 2026-10-09

**Authors:** Mengyan Zhu, Lifang Hu

**Affiliations:** ^1^ Department of Dermatology Hangzhou Third People’s Hospital Hangzhou Zhejiang China hz3yy.com

**Keywords:** androgen, androgenetic alopecia, coexistence, microinflammation, sebaceous gland, seborrheic dermatitis, synergistic therapy

## Abstract

Androgenetic alopecia (AGA) and seborrheic dermatitis (SD) are two common scalp disorders that frequently impair the quality of life and may coexist in a subset of patients across all genders. SD presents in 40%–50% of all patients diagnosed with AGA. Although these conditions are traditionally discussed separately, increasing evidence suggests that they may share overlapping pathogenic factors, including androgen‐driven sebum hypersecretion, imbalance of the scalp microecology, barrier dysfunction, and inflammatory signaling. We propose an integrated framework for understanding SD–AGA coexistence, emphasizing the interplay among endocrine influences, sebum alterations, microbial imbalance, and inflammatory activation. Therefore, the management of patients with coexisting AGA and SD should move beyond single‐target approaches and emphasize the restoration of scalp homeostasis, control of inflammatory cascades, and integration of antiandrogen therapy through comprehensive, individualized treatment strategies. This review may offer a new perspective for the combined management of these dual scalp disorders.

## 1. Introduction

Seborrheic dermatitis (SD) is a common, recurrent, chronic inflammatory skin disease that predominantly affects sebaceous gland‐rich areas, particularly the scalp, and is characterized by yellow‐red patches covered with greasy, yellowish scales or crusts and varying degrees of pruritus. Androgenetic alopecia (AGA), formerly known as seborrheic alopecia, is a progressive androgen‐dependent disorder characterized by hair follicle miniaturization with a typical patterned distribution. Although SD and AGA are distinct conditions, both exhibit high prevalence and incidence rates, with a predilection for young adults and older men [1, 2]. Both conditions significantly impair patients’ quality of life and mental health. The coexistence of AGA and SD is frequently observed in clinical practice and has attracted growing attention. However, coexistence does not necessarily establish a direct causal relationship, and the mechanistic basis remains incompletely understood.

In this review, we aim to systematically summarize the clinical characteristics and pathogenesis of both diseases across all genders, evaluate the available scientific evidence, and propose an integrated mechanism for their coexistence. This could potentially be beneficial in selecting a more appropriate systemic treatment plan from the comprehensive perspective of the scalp.

## 2. Clinical and Epidemiological Overlap Between SD and AGA

Both SD and AGA have a chronic course and require long‐term maintenance management. Under dermoscopy, AGA shows hair shaft diameter variability, increased vellus hairs, and a higher proportion of single hair follicular units. In scalp SD, scales are particularly prominent around hair follicles, accompanied by thin arborizing vessels [3]. Although AGA has traditionally been considered a noninflammatory disease, increasing evidence suggests the presence of inflammatory responses within the pilosebaceous unit [4]. Skin biopsy findings in both conditions demonstrate infiltration of inflammatory cells, particularly lymphocytes [5, 6].

In a study by Jang et al. involving 1218 patients with AGA, SD was the most common concomitant disease in both sexes, with a prevalence of 51.2% in men and 45.7% in women [7]. Similarly, SD has been identified as the most frequent associated condition in adolescents with AGA [8]. Another study reported an SD prevalence of 42.1% among patients with AGA, with both prevalence and severity scores increasing in parallel with AGA severity [9]. Although alopecia is not a typical manifestation of SD, accumulating evidence suggests that chronic inflammation in SD may be associated with an increased risk of telogen effluvium [10–12]. While SD may not directly cause hair loss, it can contribute indirectly through persistent scaling and mechanical damage from pruritus‐related scratching [13]. In some cases, hair loss may reflect poorly controlled SD [13]. However, data on the prevalence and severity of AGA in patients with SD remain limited, and the temporal relationship between the onset of SD and AGA requires further investigation. This will be more conducive to proving bidirectional causation.

Both SD and AGA show a higher prevalence in males than in females, with prevalence increasing markedly after puberty, except for infantile SD [14–17]. No significant ethnic differences in SD prevalence have been observed [18]. However, the prevalence of dandruff varies and, similar to AGA, is higher in White populations than in Asian populations [16, 17]. Shared risk factors such as obesity, high‐fat and oil‐rich diets, excessive alcohol intake, smoking, and psychological stress can aggravate both conditions [19–25]. Accumulating epidemiological and clinical evidence suggests a correlation between SD and AGA, with overlapping affected populations, suggesting a shared pathogenic background.

## 3. Mechanism of Association Between SD and AGA

### 3.1. Dual Pathological Effects of Sebaceous Gland Hyperactivity and Lipid Composition Changes

The peak prevalence of SD coincides with the period of maximal sebaceous gland activity, and lesions predominantly occur in sebaceous gland‐rich areas [26]. Patients with SD exhibit increased skin surface lipid levels compared with healthy controls, along with altered lipid composition [27, 28]. Similarly, studies in AGA mouse models have demonstrated hair follicle miniaturization accompanied by sebaceous gland hyperplasia. In individuals with alopecia, sebaceous lobules in the frontal scalp are enlarged relative to those in the normal frontal scalp, and the follicular canal is narrowed [29]. These findings suggest a potential association of both SD and AGA with sebaceous gland hyperactivity and altered sebum dynamics. In patients with AGA, the co‐occurrence of SD may be related to narrowing of the follicular canal and altered sebum drainage, which could favor sebum retention and create conditions conducive to microbial overgrowth and inflammation [30]. Therefore, sebaceous gland hyperplasia may contribute strongly to alopecia progression.

### 3.2. Microecological Imbalance: Synergy Between *Malassezia* and Major Pathogenic Bacteria

Human skin harbors more than 1000 species of microorganisms, forming a complex and dynamic skin microecological system. Among them, *Malassezia* yeasts commonly colonize healthy skin and preferentially inhabit hair follicles and sebaceous gland‐rich areas. *Malassezia* is currently considered the primary microbial factor involved in the pathogenesis of SD. Under favorable conditions, *Malassezia* secretes lipases that generate free fatty acids (FFAs), such as oleic acid and arachidonic acid, as well as lipid peroxides (LPOs) [31, 32]. Sebum provides lipid substrates for microbial growth, whereas microbial metabolites may alter local lipid composition, activate keratinocytes and immune cells, and thereby could contribute to inflammation and skin barrier dysfunction. Microbiome studies have shown that SD lesions are characterized by increased abundance of *Acinetobacter*, *Staphylococcus*, and *Streptococcus*, overgrowth of *Candida* and *Aspergillus*, and reduced levels of *Cutibacterium* species. Effective treatment is associated with a decrease in *Malassezia*, indicating that these microbial alterations are unfavorable for maintaining skin microbial homeostasis [33–37]. Antifungal agents, antibacterial agents, glucocorticoids, and calcineurin inhibitors are commonly used in the treatment of SD, with antifungal agents regarded as first‐line therapy [36], further supporting the pivotal role of the scalp microbial environment in SD pathogenesis.

The involvement of microorganisms in AGA has also attracted increasing attention. Alterations involving *Cutibacterium acnes*, *Staphylococcus*, *Corynebacterium*, and *Malassezia* have been reported in AGA [38–40]. Consistent with the observations for SD, the abundance of *Cutibacterium acnes* decreases while the abundance of the other three genera increases, indicating that the microbial signature of AGA partially overlaps with that of SD. As hair loss progresses, an increased presence of nonresident bacteria has been observed [41]. As a key component of skin microecology, microbial communities play an essential role in maintaining scalp and hair follicle health, and their imbalance may contribute to the development and progression of SD and may also influence the scalp microenvironment in AGA. In addition, Toll‐like receptors of the host recognize the membrane components on the surface of microorganisms, thereby initiating downstream signal transduction cascades that lead to the activation of NF‐κB, interferon regulatory factors, and subsequent upregulation of proinflammatory cytokines and immune mediators [42]. Therefore, alterations in the abundance or composition of even a single microbial species could exert effects on the overall microbiota equilibrium and local inflammatory milieu of the scalp and hair follicles.

### 3.3. Common Endocrine Basis: Regulation of the Androgen Metabolic Pathway

The important role of androgens in AGA was demonstrated by Hamilton in 1941 [43]. Individuals castrated before puberty or with hypogonadism do not develop AGA, whereas hair loss can occur following androgen administration [43, 44]. Testosterone, the principal circulating androgen, is converted into dihydrotestosterone (DHT) by 5α reductase within the pilosebaceous unit. DHT subsequently binds to the androgen receptor (AR) in hair follicle cells and is thought to promote shortening of the hair cycle and a premature transition from the anagen phase to the catagen phase [45]. Elevated serum DHT levels have also been detected in female patients with AGA, further supporting the role of androgens in AGA pathogenesis [46, 47].

Androgens are also thought to contribute to SD, primarily by regulating sebaceous gland activity and sebum secretion [48]. DHT binds to AR in sebaceous gland cells, activating downstream signaling pathways that promote sebocyte proliferation and differentiation and enhance sebum synthesis and secretion [49]. Sebum lipids may provide a favorable environment for the proliferation of microorganisms such as *Malassezia* and the generation of proinflammatory mediators, thereby potentially contributing to SD. The Wnt/β‐catenin signaling pathway is a key regulator of hair follicle cell development during hair morphogenesis and regeneration [50]. DHT has been shown to inhibit this pathway by upregulating glycogen synthase kinase‐3 β, leading to β‐catenin ubiquitination and degradation; this mechanism may contribute to the pathogenesis of AGA [51]. Reduced activity of the Wnt/β‐catenin signaling pathway further exacerbates inflammatory responses [52]. The onset of SD during adolescence and its higher prevalence in males further suggest androgen involvement in the regulation of sebaceous glands within hair follicles [31, 49]. However, data on circulating androgen and DHT levels in the sebaceous glands of patients with SD are still lacking. SD may involve overlapping endocrine influences although direct endocrine evidence remains limited.

### 3.4. From “Microinflammation” to Chronic Inflammation: The Influence of the Immune Cascade Reaction on the Hair Follicle Cycle

The inflammatory response in AGA is characterized by a slow, subtle process known as microinflammation, which is closely associated with progressive hair follicle miniaturization [53]. Histopathological studies have demonstrated lymphocyte infiltration, activated T cells, and mast cell degranulation in the hair follicle infundibulum of patients with AGA [54]. These inflammatory changes may directly or indirectly impair the cyclic regenerative capacity of hair follicle stem cells. By releasing various cytokines, they participate in follicular miniaturization [5]. SD is a chronic inflammatory skin disorder strongly associated with *Malassezia*. Under favorable conditions, *Malassezia* secretes lipases that generate fatty acids such as oleic acid and arachidonic acid, as well as LPOs. These metabolites have been reported to promote dendritic cell maturation and activate Th2 and Th17 cells, thereby potentially triggering inflammatory signaling pathways and the release of multiple proinflammatory cytokines, including IL‐17, IL‐2, and IFN‐γ, leading to sustained inflammation and skin barrier disruption [31, 31, 55]. This chronic low‐grade inflammatory state, in combination with DHT, may accelerate hair follicle miniaturization and help sustain a self‐perpetuating interaction between hair loss and scalp barrier dysfunction.

### 3.5. The Potential Association Between Genetic Susceptibility and Genetic Polymorphisms

AGA is a polygenic disorder with strong familial aggregation. Evidence from the UK Biobank and twin studies has confirmed that the heritability of AGA in males is estimated at 62%–80% [56, 57]. Biobank‐scale genome‐wide association study has identified multiple susceptibility loci for AGA, pinpointing 71 independent risk loci that collectively account for approximately 38% of the phenotypic variance [58]. Leading risk signals include the AR, EDA2R, alongside genes involved in the WNT signaling pathway [58]. These loci participate in androgen metabolism, the regulation of hair follicle development, and dermal intercellular communication. Genetic factors are thought to influence individual hair follicle sensitivity to androgens and resistance to miniaturization, thereby influencing the age of onset, progression rate, and final severity of hair loss in patients with AGA and accounting for interindividual variability [59].

SD is also associated with genetic factors. To date, more than 10 gene mutations or protein deficiencies have been identified that can induce SD or SD‐like eruptions [60]. For example, mutations in TRAF3IP2 (ACT1) can impair skin immune responses by disrupting IL‐17 signaling, leading to SD like inflammatory reactions against cutaneous fungi [61]. Although familial aggregation has been observed in SD, its genetic predisposition remains unclear. Nevertheless, a preliminary genome‐wide association study identified two genome‐level susceptibility loci associated with SD, suggesting a potential role of genetic susceptibility in its pathogenesis [62]. At present, there is no convincing evidence for shared risk locus, genetic correlation, or Mendelian random‐derived causal links between SD and AGA. Future genetic analyses testing these relationships would provide a feasible approach to validate the central hypothesis linking the two conditions.

## 4. Integrated Mechanism of SD–AGA Coexistence

Based on the aforementioned information, we have summarized the coexistence mechanism between SD and AGA. Sebaceous dysregulation, microbial imbalance, androgen‐related signaling, inflammation, and genetic susceptibility appear to represent overlapping biological domains between SD and AGA. At the pathophysiological level, SD and AGA exhibit notable interactions, and androgen responsiveness is more plausibly considered primary drivers, particularly for AGA and possibly for part of the SD‐prone scalp phenotype. Circulating testosterone is converted to DHT by 5α‐reductase, which simultaneously promotes hair follicle miniaturization, sebaceous gland hyperplasia, and inflammatory responses through multiple pathways [45, 48].

At intermediate levels, increased lipid secretion leads to *Malassezia* reproduction and altered lipid composition, microbial metabolites, and epidermal barrier disruption may activate innate immune pathways, including downstream cytokine networks [30–32]. These events may amplify local inflammation and further disrupt scalp barrier.

At the downstream level, persistent perifollicular microinflammation may aggravate SD symptoms while also contributing to a follicular microenvironment unfavorable for normal hair cycling, thereby potentially accelerating AGA progression (see Figure 1). However, current evidence primarily supports association rather than direct causation, and these proposed links require validation in well‐designed human mechanistic and longitudinal studies. Current data more strongly support the notion that dysregulation in endocrine, sebaceous, microbial, epidermal barrier, and inflammatory pathways—mediated by their dynamic interplay—synergistically elevates the risk of coexistence.

**FIGURE 1:**
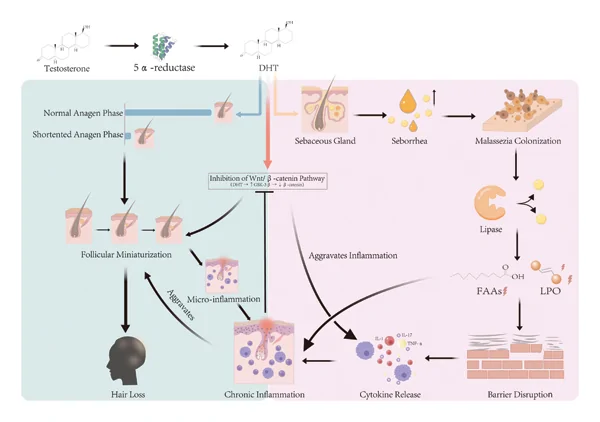
Coexistence between seborrheic dermatitis (SD) and androgenetic alopecia (AGA). Androgens are converted to dihydrotestosterone (DHT), which induces hair follicle miniaturization, sebaceous gland hyperplasia, and pilosebaceous inflammation. Excess sebum promotes *Malassezia* growth, leading to the production of inflammatory mediators that disrupt the scalp barrier. Chronic inflammation synergizes with DHT signaling, accelerating follicular fibrosis and hair loss and forming a self‐reinforcing pathological cycle (FAAs, free fatty acids; LPO, lipid hydroperoxide).

## 5. Comprehensive Treatment Strategies Based on Target Synergy and Prospects

At present, no formal guideline specifically addresses the treatment of patients with concurrent SD and AGA. As a result, current combination approaches in clinical practice are extrapolated from interventions established for SD or AGA individually rather than from trials conducted in the coexisting population.

According to international guidelines [63], current treatment for AGA primarily focuses on inhibiting 5α‐reductase activity or blocking the AR. Finasteride is a selective inhibitor of type 2 5α‐reductase and is widely recognized as the first‐line oral pharmacotherapy for male AGA. It exerts its therapeutic effect primarily through sustained reduction of serum DHT levels, thereby attenuating progressive hair follicle miniaturization. Dutasteride, another 5α‐reductase inhibitor, inhibits both type 1 and type 2 5α‐reductase isoenzymes and can serve as an alternative to finasteride. Systemic 5α‐reductase inhibitors are not routinely recommended for the management of female AGA. Oral antiandrogens, including spironolactone and cyproterone acetate, are evidence‐informed alternatives. Topical minoxidil is another cornerstone first‐line therapy, while low‐level laser therapy, stem cell–based products, platelet‐rich plasma, and hair transplantation may be considered in selected patients. Combination therapy with microneedling can improve percutaneous penetration, thereby enhancing the efficacy of topical minoxidil for AGA [64]. Novel therapies such as oral minoxidil, topical finasteride, topical spironolactone, botulinum toxin, stem cell therapy, exosomes, nutritional supplement, PGE2 analogs, adenosine, and caffeine offer alternatives to standard of care therapies for patients [65–67].

In contrast, the management of SD emphasizes anti‐inflammatory, antifungal, and skin barrier–regulation strategies [68]. Commonly used topical agents include azole antifungals, glucocorticoids, and salicylic acid. Expanded topical treatment options include antifungal and keratolytic topical agents (piroctone olamine, coal tar, selenium sulfide, and sodium sulfacetamide) and soothing and barrier‐supporting topical preparations (bisabolol, glycerin, telmesteine, lithium gluconate/succinate, glycerin, and aloe vera), while mud therapy and phototherapy serve as complementary physical interventions [69, 70]. New treatments, including PDE4 inhibitors (roflumilast, crisaborole, and apramilast), topical and oral JAK inhibitors, probiotics, and microbiota‐targeted therapy, provide new hope for severe and refractory SD [71].

Although the primary therapeutic targets of AGA and SD differ, combined regulation of the scalp microenvironment may enhance treatment outcomes in patients with coexisting AGA and SD. Antifungal therapy not only relieves pruritus and desquamation associated with SD but also indirectly reduces perifollicular inflammation by decreasing *Malassezia*. Both animal studies and clinical observations have shown that topical ketoconazole increases the area of hair regrowth, enlarges hair shaft diameter, and improves the hair follicle index compared with controls, suggesting its potential as an adjunct or alternative treatment for AGA [72]. In addition, antidandruff shampoos may help reduce hair loss. One study reported that daily use of a 1% zinc pyrithione shampoo for 26 weeks resulted in moderate and sustained improvement in hair growth [73]. Drugs with both anti‐inflammatory and microbiota‐regulating effects, such as topical traditional Chinese herbal formulations, can help treat SD and relieve alopecia [74]. Local antiandrogen therapy, including topical antiandrogens and 5‐alpha reductase inhibitors, can suppress sebaceous gland activity and reduce sebum production [75]. This reduction in sebum output represents a mechanistic link between AGA and SD, as excess sebum provides a favorable microenvironment for *Malassezia* overgrowth and scalp inflammation. Nevertheless, robust clinical evidence supporting the direct therapeutic benefit of these agents for SD remains limited.

In patients with coexisting disease, particularly in patients with chronic inflammation and excessive sebum production, concurrent targeting both inflammatory and androgen pathways concurrently may provide synergistic therapeutic benefits. The following treatment‐sequencing considerations represent author‐derived clinical inference, given the absence of dedicated clinical trials in this comorbid population. In individuals with mild AGA and severe SD, priority should be given to SD‐directed control. First‐line SD‐targeted measures include azole antifungals and short‐course topical glucocorticoids. Once scalp inflammation and desquamation are well controlled, minoxidil‐based AGA therapy can be initiated, and propylene‐glycol‐free minoxidil foam should be preferred to minimize irritant risk. For patients with severe AGA and mild SD, AGA‐directed therapy can be started upfront. First‐line options include oral finasteride (male patients) together with topical minoxidil. For female patients, spironolactone combined with minoxidil is a reasonable alternative. These androgen‐modulating therapies that reduce sebum production may lead to improvement or even remission of SD. Other therapies, including low‐energy laser irradiation, scalp salicylic acid scraping, topical herbal extracts (such as bisabolol), platelet‐rich plasma, stem cell–based products, and JAK inhibitors, targeting both inflammation and hair follicles, also show promise for dual regulation.

There is currently no clear treatment hierarchy for SD–AGA coexistence, and high‐quality randomized controlled trials specifically evaluating coexisting patients are lacking. Many therapies may offer future advantages for SD–AGA coexistence by targeting shared pathogenic pathways rather than managing each disorder in isolation. Although most of these approaches remain investigational, they are of increasing interest because they address the scalp ecosystem more comprehensively.

In conclusion, although AGA and SD present with distinct clinical manifestations, substantial interactions exist between the two conditions regarding affected populations and underlying pathogenic mechanisms. Future studies should further explore immune–endocrine interactions in the context of AGA and SD coexistence, the dynamic balance among androgens, sebaceous glands, the cutaneous microbial flora, and the regulatory effects of inflammatory signaling pathways on the hair follicle cycle. For patients affected by both disorders, a multifaceted, staged, and individualized treatment strategy should be advocated, aiming to achieve improved therapeutic outcomes through a comprehensive approach of anti‐inflammation, oil control, regulation of microbiota, and androgen inhibition.

## Author Contributions

Mengyan Zhu performed conception and design of research, analyzed data, prepared figures and tables, and drafted the manuscript. Lifang Hu edited and revised the manuscript and approved the final version of the manuscript.

## Funding

This study was funded by the Chinese Medicine Research Program of Zhejiang Province, 2024ZL728; Hangzhou bio‐medicine and health industry development support science and technology project, 2024WJC139; and the Construction Fund of Key Medical Disciplines of Hangzhou, 2025HZGF07.

## Conflicts of Interest

The authors declare no conflicts of interest.

## Data Availability

Data sharing is not applicable to this article as no datasets were generated or analyzed during the current study.
